# A Consensus on Immunization Recommendations for Healthcare and Other Workers at the Tokyo 2020 Olympic and Paralympic Games: A Delphi Survey

**DOI:** 10.31662/jmaj.2018-0012

**Published:** 2019-02-01

**Authors:** Shoichi Shimizu, Koji Wada, Shinji Fukushima

**Affiliations:** 1 Department of Immunology and Parasitology, University of Occupational and Environmental Health, Fukuoka, Japan; 2 Health Promotion Center, Mitsubishi Electric Corporation Kamakura Works, Kamakura, Japan; 3 Department of Public Health, International University of Health and Welfare, Tokyo, Japan; 4 The Research Group on Occupational Health for Health Care Workers, Japan Society for Occupational Health, Tokyo, Japan; 5 Travellers’ Medical Center, Tokyo Medical University Hospital, Tokyo, Japan

**Keywords:** Delphi Technique, Health Personnel, Immunization, Japan, Olympics

## Abstract

This study aimed to build a consensus on recommendations of immunity requirements for vaccine-preventable diseases among healthcare and non-healthcare workers, including volunteers, at the Tokyo 2020 Olympic and Paralympic Games. We used a two-round Delphi method with a group of 17 Japanese medical doctors involved in vaccination or public health administration. We asked them to rank the importance of immunity to each vaccine-preventable disease as mandatory, recommended, considered if possible, or standard precautions only. The response rate was 88.2% (15/17) for the first questionnaire and 100% (17/17) for the second. All respondents considered that immunity to measles, rubella, varicella, mumps, and hepatitis B should be mandatory for healthcare workers, and 15 of 17 respondents considered that immunity to influenza should also be mandatory. Seven, three, two, and two respondents thought that immunity to pertussis, meningococcal disease, diphtheria, and tetanus should be mandatory, and ten, 11, seven, and seven thought it should be recommended. For non-healthcare workers, immunity to measles, rubella, and varicella was considered mandatory by 17, 15, and 15 respondents. Ten and eight respondents thought that immunity to mumps and influenza should be mandatory, and seven thought that it should be recommended. In conclusion, the consensus was that immunity to measles, rubella, and varicella should be mandatory for both healthcare and non-healthcare workers. Immunity to mumps, hepatitis B, and influenza was also considered mandatory for healthcare workers. Further discussions may be needed to develop a consensus on other vaccine-preventable diseases, especially if vaccination is not routine for adolescents or adults in Japan.

## Introduction

International mass gatherings may increase the risk of an infectious disease outbreak. It has therefore been suggested that precautions be taken against vaccine-preventable diseases (VPDs) before mass gatherings ^(1)^. The Olympic Games are among the busiest events held worldwide. Although experience suggests that the incidence of a major infectious disease outbreak is typically low or similar to non-Olympic periods, outbreaks of influenza and other diseases have been reported. Several VPDs, including hepatitis B, pertussis, meningococcal disease, varicella, mumps, and typhoid fever were reported during the 2004 Olympic Games in Athens.

A prospective study on influenza revealed that the organizing committee volunteers and staff accounted for the largest number of diagnosed cases of influenza during the 2002 Salt Lake City Winter Olympics. Although there have been recommendations for visitors to mass gatherings to prevent infectious diseases ^(2)^, there have been no recommendations for workers and volunteers at the Olympic Games or other mass gatherings.

Outbreaks of measles, rubella, and other airborne VPDs have been reported in Japan outside mass gatherings. An epidemiological survey revealed that the workplace was the most common location for rubella transmission in Japan. Healthcare workers are encouraged to prevent occupationally acquired infections, because of their contact with patients and infective material, and several organizations have published recommendations on the vaccination of healthcare workers.

Immunization recommendations for the Tokyo 2020 Olympic Games are therefore needed urgently for both healthcare and other workers to reduce the risk of VPDs. This study aimed to build a consensus on recommendations for immunity requirements for VPDs among healthcare and non-healthcare workers, including volunteers, at the Tokyo 2020 Olympic and Paralympic Games.

## Materials and Methods

We used the Delphi method, which is a structured group communication process used to attain a reliable group consensus and make decisions based on expert judgment ^(3)^. We conducted a Google Forms–based anonymous and multiple-choice online survey on the importance of immunity to each VPD for healthcare and non-healthcare workers at the Tokyo 2020 Olympics.

We recruited 17 Japanese medical doctors involved in practical vaccination or public health administration. We defined healthcare workers as medical doctors and nurses who would undertake initial medical examinations, and non-healthcare workers as all others who would work or volunteer at the site of the Tokyo 2020 Olympics. We asked participants to rank each VPD against four degrees of importance: mandatory (it should be mandatory for every worker to be immune), recommended (immunity is not mandatory but is recommended), considered if possible, and not necessary (standard precautions only). We selected the VPDs based on the Japanese Immunization Act on routine vaccinations, and they were diphtheria, pertussis, poliomyelitis, measles, rubella, Japanese encephalitis, tetanus, *Haemophilus influenzae* type b, pneumococcal disease, varicella, hepatitis B, and influenza. We also included additional VPDs for which immunization is mandatory in other countries, including mumps and meningococcal disease, or recommended for travelers, including hepatitis A, typhoid fever, cholera, and rabies. We asked the participants not to consider the cost of vaccination and the supply system, but to simply assess the priority based on the efficiency of vaccination.

We sent the first questionnaire by email at the beginning of June 2017. We sent the second questionnaire, which was the same questions with feedback on the results of the first questionnaire, at the end of June 2017.

Our data were anonymized, and individual participants could not be identified. There was no explicit written consent for each participant, but we assumed that completing the round 1 online survey implied consent to participate. We conducted a Delphi study involving only healthcare professionals. Based on the existing ethical guidelines for medical and health research in Japan, ethical approval for this study was waived by the ethics committee of the National Centre for Global Health and Medicine, Japan (application number 2476).

## Results

The response rate was 88.2% (15/17) for the first questionnaire, and 100% (17/17) for the second. The results of the second survey are shown in Table 1 for healthcare workers, and in Table 2 for non-healthcare workers. Over half of the respondents agreed that immunity was mandatory or recommended for healthcare workers for ten VPDs: measles, rubella, varicella, mumps, hepatitis B, influenza, pertussis, meningococcal disease, diphtheria, and tetanus (Table 1).

**Table 1.** Number of Responses for Each Level of Priority, by Disease, for Healthcare Workers at the Tokyo 2020 Olympic and Paralympic Games.

	Mandatory for all	Not mandatory but recommended	Considered if possible	Not necessary
Measles	17	0	0	0
Rubella	17	0	0	0
Varicella	17	0	0	0
Mumps	17	0	0	0
Hepatitis B	17	0	0	0
Influenza	15	2	0	0
Pertussis	7	10	0	0
Meningococcal disease	3	11	3	0
Diphtheria	2	7	6	2
Tetanus	2	7	5	3
Poliomyelitis	1	3	10	3
Japanese encephalitis	1	4	6	6
Hepatitis A	1	3	7	6
Typhoid fever	1	1	6	9
Pneumococcal disease	0	2	6	9
Cholera	0	0	5	12
Rabies	0	0	4	13
*Haemophilus influenzae* type b	0	0	3	14

**Table 2.** Number of Responses for Each Level of Priority, by Disease, for Non-healthcare Workers at the Tokyo 2020 Olympic and Paralympic Games.

	Mandatory for all	Not mandatory but recommended	Considered if possible	Not necessary
Measles	17	0	0	0
Rubella	15	2	0	0
Varicella	15	2	0	0
Mumps	10	7	0	0
Influenza	8	7	2	0
Pertussis	2	11	3	1
Hepatitis B	1	8	5	3
Meningococcal disease	1	6	8	2
Tetanus	0	10	2	5
Diphtheria	0	8	5	4
Poliomyelitis	0	6	5	6
Japanese encephalitis	0	5	6	6
Hepatitis A	0	1	9	7
Pneumococcal disease	0	1	8	8
Typhoid fever	0	1	6	10
*Haemophilus influenzae* type b	0	1	4	12
Cholera	0	0	4	13
Rabies	0	0	4	13

All respondents considered that healthcare workers needed immunity to measles, rubella, varicella, mumps, and hepatitis B. Nearly all (15 of 17) considered it should also be mandatory for influenza, with the remaining two saying that immunity was recommended. Seven respondents thought that immunity to pertussis should be mandatory, and ten thought it should be recommended.

Over half of the respondents agreed that immunity should be mandatory or recommended for non-healthcare workers for eight VPDs: measles, rubella, varicella, mumps, influenza, pertussis, hepatitis B, and tetanus. Overall, the views were that requirements should be lower for non-healthcare than for healthcare workers (Table 2). Immunity to measles, rubella, and varicella was considered mandatory by 17, 15, and 15 respondents. Views on mumps and influenza were more divided, with ten and eight considering that immunity should be mandatory, and seven and seven that it should be recommended.

## Discussion

This study built a consensus on the importance of immunity to VPDs for healthcare and other workers at the Tokyo 2020 Olympics. It used the Delphi method to explore opinions among Japanese medical doctors engaged in practical vaccination or public health administration. The study participants agreed that immunity to respiratory transmittable diseases, such as measles, rubella, and varicella should be mandatory for all workers, with immunity to mumps, hepatitis B, and influenza also required for healthcare workers. Previous recommendations have emphasized the necessity of vaccination for various situations, including mass gatherings ^(4)^, but to our knowledge, this study is the first to compare recommendations on immunization for healthcare and non-healthcare workers for mass gatherings in Japan.

There were some differences between healthcare and other workers. Most participants agreed that there was a greater need for healthcare workers to be immune to hepatitis B, pertussis, and meningococcal disease. The overall requirements were higher for healthcare workers than for others. This may be because they have a higher risk of exposure to infectious diseases or their absence would have a greater impact ^(5)^ than non-healthcare workers. A previous study found that healthcare workers at the Olympic Games responded primarily to patients with infectious diseases. A seroprevalence survey among Japanese healthcare workers suggested that there was a possibility of measles, rubella, and mumps outbreaks in particular age or sex groups ^(6)^. Vaccinations against measles, rubella, varicella, meningococcal disease, influenza, diphtheria, and hepatitis B are recommended for healthcare workers by the World Health Organization and U.S. Centers for Disease Control and Prevention.

Our results for non-healthcare workers are comparable to the health advice given to travelers to the Rio 2016 Olympic Games, except for travel-related VPDs such as hepatitis A, typhoid fever, rabies, and yellow fever, where the recent incidence in Japan has been low or none. These workers may come into contact with patients with an infectious disease, resulting in its spread.

Immunity to pertussis was considered relatively important for both groups in this study, especially for healthcare workers, despite the absence of an approved pertussis vaccine, such as the Tdap (tetanus, diphtheria, and pertussis) booster vaccine, for adolescents or adults in Japan. There have been recent epidemics of pertussis among adolescents and adults, so it may be necessary to consider introducing an approved pertussis vaccine for adolescents or adults, as has been done in Western countries. Immunity to meningococcal disease was also recommended for healthcare workers in this study, although a vaccine against meningococcal disease is not routine in Japan. This recommendation could be considered in light of previous epidemics following mass gatherings and recommendations in other countries ^(2)^.

There would be problems to overcome in delivering these recommendations. The first would be the vaccine cost burden: in other words, who is responsible for the cost of the vaccine? All vaccinations except routine vaccines for children or older people are paid for by the individual in Japan. It is hard to enforce vaccination since the cost may be prohibitive for individuals. Financial assistance from the employing organization would be desirable. The second problem would be vaccine supply. Vaccines against meningococcal disease and mumps have been approved, but are not routinely given in Japan, and are therefore sometimes difficult to obtain.

This study had limitations. We did not provide any additional information that could affect the participants’ suggestions on the prioritized vaccination. We expected our participants to suggest the necessity of vaccination based on their background and expertise. Second, further discussions and investigations based on the latest situations, such as the prevalence of VPDs, could be needed under the initiative of the organizing committee of the Tokyo 2020 Olympic and Paralympic Games.

In conclusion, this study suggests that vaccination professionals consider that all workers at the Tokyo 2020 Olympics should be immune to some VPDs, particularly measles, rubella, and varicella, and that immunity to mumps, hepatitis B, and influenza is also essential for healthcare workers. Further discussions may be needed to develop recommendations on other VPDs, such as meningococcal disease and pertussis. Neither of these is part of a routine vaccination program for adolescents and adults in Japan, although outbreaks have been reported at mass gatherings.

## Article Information

### Conflicts of Interest

None

### Sources of Funding

This work was supported by the National Center for Global Health and Medicine (27-4) and the Research Group on Occupational Health for Health Care Workers, Japan Society for Occupational Health, Japan.

### Acknowledgement

The authors express their gratitude to all the experts who participated in this survey.

### Author Contributions

SS and KW conceived the study design and analyzed the data. SF and KW assisted in testing questionnaires and conducted feedback meetings. SS, SF, and KW wrote the manuscript.

### Approval code

Ethical approval for this study was waived by the ethics committee of the National Centre for Global Health and Medicine, Japan.
